# Acute Respiratory Distress Syndrome: Role of Oleic Acid-Triggered Lung Injury and Inflammation

**DOI:** 10.1155/2015/260465

**Published:** 2015-11-12

**Authors:** Cassiano Felippe Gonçalves-de-Albuquerque, Adriana Ribeiro Silva, Patrícia Burth, Mauro Velho Castro-Faria, Hugo Caire Castro-Faria-Neto

**Affiliations:** ^1^Laboratório de Imunofarmacologia, Instituto Oswaldo Cruz (FIOCRUZ), 21040-900 Rio de Janeiro, RJ, Brazil; ^2^Departamento de Biologia Celular e Molecular, Instituto de Biologia, Universidade Federal Fluminense, 24020-15 Niterói, RJ, Brazil; ^3^Departamento de Medicina Interna, Faculdade de Ciências Médicas, Universidade do Estado do Rio de Janeiro, 20550-900 Rio de Janeiro, RJ, Brazil

## Abstract

Lung injury especially acute respiratory distress syndrome (ARDS) can be triggered by diverse stimuli, including fatty acids and microbes. ARDS affects thousands of people worldwide each year, presenting high mortality rate and having an economic impact. One of the hallmarks of lung injury is edema formation with alveoli flooding. Animal models are used to study lung injury. Oleic acid-induced lung injury is a widely used model resembling the human disease. The oleic acid has been linked to metabolic and inflammatory diseases; here we focus on lung injury. Firstly, we briefly discuss ARDS and secondly we address the mechanisms by which oleic acid triggers lung injury and inflammation.

## 1. Introduction

Despite advances in the past decades in the knowledge and treatment of acute respiratory distress syndrome (ARDS) the mortality remains unacceptably high [1], ranging from 27% to 45% [2]. Here we discuss lung injury and inflammation mechanisms induced by a single fatty acid molecule, the oleic acid. For that, we focus on mechanisms of cell death, edema formation and alveoli swelling, cell and intracellular signaling activation pathways, and inflammatory mediator production that would lead to severe lung damage and loss of function.

## 2. Acute Respiratory Distress Syndrome

The lung is the primary target of diverse insults including, but not limited to, microbe's infection, pollutants, toxic gasses, gastric acids, autoantibodies, fatty emboli, and free fatty acids (Table 1). Initial lung injury can evolve to a severe disease known as acute respiratory distress syndrome (ARDS) [3]. One of the initial steps of this syndrome is the edema formation followed by an intense inflammatory response causing lung functional and structural damage and patients with ARDS demand intensive care treatment.

**Figure 1:**
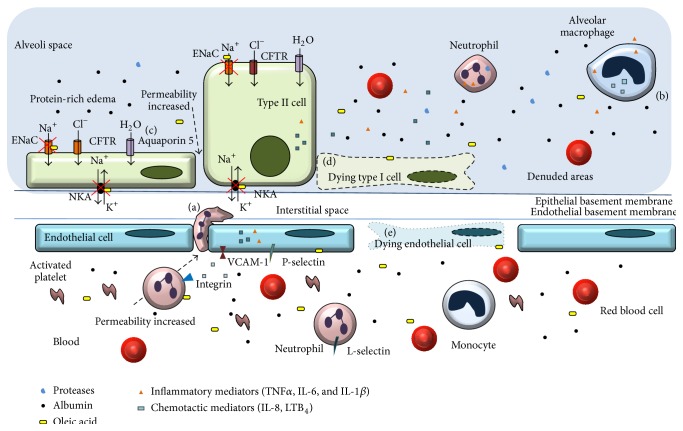
Effects of oleic acid in acute respiratory distress syndrome. Oleic acid induces damage in epithelial and endothelial cells, with increased permeability and protein-rich edema, with denuded areas in alveoli forming a hyaline membrane. Oleic acid induces apoptosis or necrosis in alveolar type I or type II cells (d), depending on the insult origin. Alveolar macrophages (b) act as sentinels triggering the immune response and produce chemotactic and inflammatory mediators. Chemoattractant mediators produced by alveolar macrophages and epithelial and endothelial cells induce increased adhesion molecules such as VCAM-1, selectins, and integrins, favoring the inflammatory cell infiltration. Neutrophils (a) are first cells migrating to lung and their excessive recruitment contributes to the lung pathology and they produce and release other inflammatory mediators and other molecules such as proteases and elastase. Aquaporin 5 is (c) a water channel responsible for moving water from the alveoli to the lung interstitium, AO-induced lung injury could advent via intrapulmonary or extrapulmonary. In case of extrapulmonary ARDS the main target will be endothelial cells and leukocytes inducing endothelial cell death (e). Similar to humans, OA induces lung hemorrhage. OA inhibits ENaC and NKA inducing and/or avoiding edema fluid clearance. ENaC: epithelial sodium channel; CFTR: cystic fibrosis transmembrane conductance regulator; NKA: Na/K-ATPase; VCAM-1: vascular cell adhesion molecule 1.

The epithelial cell lining in the alveoli-endothelial barrier produces a thin liquid layer containing secreted peptides and proteins contributing to host defense and preserving the primary lung function of transporting CO_2_ from the blood to alveoli and O_2_ from the alveoli to the bloodstream (Table 2). Alveoli are the functional lung unit, and they are covered by alveolar type I and type II cells (Figure 1). Alveolar type I cells comprise approximately 90% of the alveolar epithelium, and the remaining 10% is formed by cuboidal type II cells that handle surfactant secretion and by epithelial cell regeneration after injuries (Table 2).

Lesions to the alveolar capillary endothelium and epithelium result in barrier disruption leading to plasma proteins leakage and edema formation characterizing the exudative phase [4]. In addition to activation of the other effectors cells such as neutrophils, macrophages, endothelium, and epithelium activation, platelets also contribute to the alveolar damage in lung injury [3]. In the acute phase, cytokines and lipids are released, leading to alveolar-capillary barrier loss and hyaline membrane formation [5].

ARDS is a syndrome comprising respiratory failure with acute hypoxemia and alveolar damage secondary to an intense lung inflammatory response to different types of insults which is not mainly due to left atrial hypertension [6]. Recently, a new definition (the Berlin definition) proposed 3 mutually exclusive categories of ARDS based on the degree of hypoxemia, mild, moderate, and severe [2], and, therefore, the term acute lung injury (ALI) is no longer used.

## 3. Inflammatory Cells and Mediators

The incidence of leukocytes together with alveolar edema, hemorrhage, and hyaline membrane formation indicates that an exaggerated inflammatory response underlies the pathogenesis of early steps of pulmonary ARDS. Recognition of danger-associated molecular patterns (DAMP) by lung epithelium and alveolar macrophages is a compelling force to induce acute lung inflammation [7] (Table 2). The unbalanced inflammatory response including leukocytes recruitment and/or their activation may damage the epithelial or endothelial layer. Neutrophils are first cells migrating to lung and their excessive recruitment contributes to the tissue damage and inflammation [8] (Figure 1(a)) because they release proteases and increase the production of reactive oxygen species and inflammatory mediators [3]. In mice, key chemotactic factors to neutrophil recruitment to the lung are the chemokine CXCL1/GRO alpha (also known as KC) and chemokine (C-X-C motif) ligand 2 (CXCL2) and CXCL5 [9]. Extracellular ATP (eATP) also plays a role in neutrophil recruitment [10]. Alveolar macrophages (Figure 1(b)) act as sentinels triggering the immune response and producing chemotactic and inflammatory mediators [3]. Thrombospondin-1, a circulating plasma glycoprotein detected in bronchoalveolar lavage fluids in ARDS patients, disrupts the endothelial barrier by tyrosine kinase-dependent phosphorylation of zonula adherens proteins [11].

Cytokines such as TNF*α* and interleukins (mainly IL-1*β* and IL-6) are important mediators in the development of ARDS, contributing to augmented vascular permeability and organ dysfunction [12]. High pulmonary edema fluid levels induced by IL-8 were associated with impaired alveolar fluid clearance in ARDS patients [13].

## 4. Lung Edema Clearance

Pulmonary edema results from a combination of both increased fluid filtration and impairment of transepithelial Na^+^ transport. Alveolar fluid clearance (AFC) is driven by sodium transport across the airway epithelium, which creates mini-osmotic gradient removing water from the alveoli driving it to the bloodstream.

This mechanism depends on the apically located epithelial sodium channel (ENaC) and the basolaterally located enzyme sodium potassium ATPase (NKA). *β*-adrenergic agonists are noticeable activators of Na^+^ channels in the alveolar epithelium improving fluid clearance and edema resolution in experimental ARDS models [14]. Moreover, impairment of the enzyme NKA during ARDS not only avoids the resolution of lung edema but also intensifies its formation. In this regard, Na^+^ transport and edema clearance are associated with better outcomes in patients with sepsis and ARDS [11].

In addition to the sodium transport, the chloride transport via the cystic fibrosis transmembrane conductance regulator (CFTR) is necessary for the AFC. In an animal model of cystic fibrosis, with the lack of cystic fibrosis transmembrane conductance regulator (CFTR), alveolar fluid clearance was decreased. Using glibenclamide (an inhibitor of potassium and CFTR channels) in* in situ* perfused and nonperfused mouse lungs and in* ex vivo* human lungs, fluid clearance was impaired [11].

Finally, water crosses the alveolar epithelium either paracellularly via tight junctions or transcellularly via aquaporins [14]. Aquaporin 5 (AQP5) is expressed on the apical surface of both cell types I and II (Figure 1(c)) and is responsible for moving water from the alveoli to the lung interstitium. A significant decrease in airway-capillary water permeability is seen in lungs of AQP5 deficient mice [15].

Besides alveolar liquid, protein excess needs to be removed from alveolar space, albumin can be taken up by alveolar epithelial cells by the multiligand receptor megalin (low-density lipoprotein endocytic receptor family), and its inhibition resulted in decreased albumin binding and uptake in monolayers of primary alveolar type II and type I cells in cultured lung cells [16].

Overall, the rate of alveolar fluid transport depends on the expression and activity of ENaC, NKA, and CFTR opening. To complete edema reabsorption ion transport, water channels, and albumin transport are also important. Therefore, endothelial and epithelial barrier integrity is essential for optimal fluid balance and cell injury and/or defects on the ion transport caused by pathogens or other damaging compounds end up in decreased AFC [6]. The lesser AFC correlates with a longer stay in the intensive care unit and increased mortality in patients.

## 5. Origin of Pulmonary Insult

The ARDS pathogenesis has been classified as pulmonary (with a direct hit on lung cells) or extrapulmonary (with an indirect hit, affecting a distant organ and leading to a systemic inflammatory response) [17]. Despite the insult applied to the lung, through airways or circulation, the final result is diffuse alveolar damage. Then, any local (e.g., pneumonia) or systemic inflammation (e.g., pancreatitis) can lead to critical lung function alterations. An extensive injury to the epithelial and endothelial cell, hyaline membrane formation, and increased amount of apoptotic neutrophils is observed in pulmonary insult. In the extrapulmonary injury mediators released from extrapulmonary locations into the blood target mainly endothelial cells, leading to microvascular congestion, endothelial cell activation, an increase in vascular permeability, and interstitial edema [18].

## 6. Oleic Acid

Oleic acid (18:1 n-9) is an unsaturated fatty acid in plants and animals [19, 20]. Oleic acid is the most common and abundant fatty acid in the body of healthy individuals. It is present in human plasma, cell membranes, and adipose tissue [21, 22]. Oleic acid not only affects membrane fluidity but also facilitates membrane docking and activity of G-protein coupled receptors (GPCR) and related signaling molecules [21].

The effects of oleic acid on cells are mediated by mechanisms such as signaling through cell surface receptors or nuclear receptors [23]. Free fatty acid receptor 1 and GPR120 are membrane GPCR activated by medium and long-chain free fatty acids, as oleic acid. Free fatty acid receptor 1 also known as FFAR1 or GPR40 [24] is expressed primarily in pancreatic beta-cells and contributes to insulin secretion. Its activation leads to an increase in the intracellular Ca^++^ concentration and activation of the extracellular signal-regulated kinase (ERK)1/2 in CHO cells [23] (Table 3).

Nonesterified fatty acids (NEFA) are carried in the bloodstream bound to albumin, thus avoiding their cytotoxicity [25]. Different cells exhibit morphological features of apoptosis and necrosis after fatty acid exposure [26]. Fatty acids also alter the membrane structure, transmembrane signaling, and cell cycle control [27, 28]. They can also modify cellular functions requiring the participation of peroxisome proliferators-activated receptors (PPAR). PPAR are nuclear receptors that regulate the lipid metabolism, inflammation, cellular growth, and differentiation [29].

Altered circulating fatty acid levels are linked to pathologies such as obesity, diabetes mellitus, coronary heart disease, atherosclerosis, and cancer [30]. More important, the severity of diseases such as sepsis, leptospirosis, pancreatitis, and preeclampsia correlates with increased serum fatty acids levels and the drop in plasma albumin concentration, suggesting fatty acid toxicity [31–33].

## 7. Oleic Acid-Induced Lung Injury and Inflammation

Oleic acid lung injury presents an early phase of necrosis and microvascular thrombosis, followed by a repair phase with the proliferation of type II cells and fibrotic foci in subpleural areas [34]. Microscopically, the injury is multifocal and heterogeneous, ranging from small edema areas to hemorrhagic infiltration with fibrin deposition [35]. The histological changes of oleic acid-induced lung injury are associated with marked functional changes. As in ARDS, lung injury after oleic acid challenge presented extravasation of fluid to the extravascular space and decreased liquid reabsorption, resulting in extravascular lung water accumulation. Pulmonary microvascular permeability is markedly increased, with extravascular lung water accumulation and leakage of protein-rich fluid into the air spaces [36] (Figure 1(d)).

**Figure 2:**
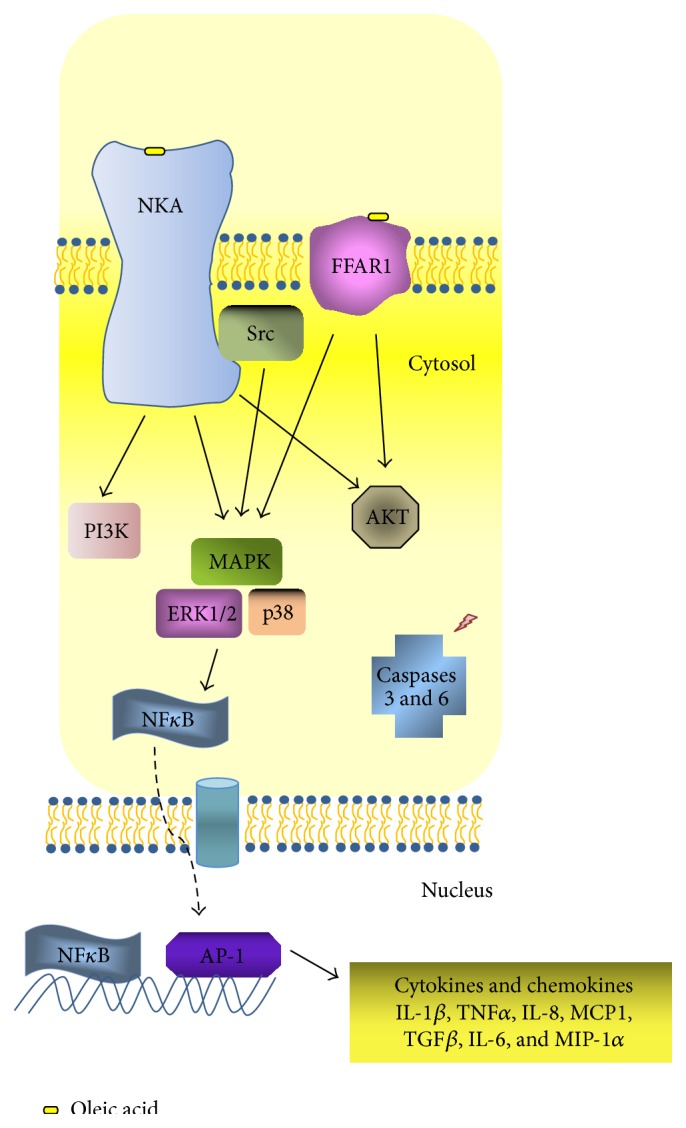
Intracellular pathways activated in oleic acid-induced lung injury and inflammation. Oleic acid triggers intracellular pathways through different receptors ending up in inflammatory mediator production and/or cell death. MAPK: mitogen-activated protein kinases, ERK1/2: extracellular signal-regulated kinases, NF*κ*B: nuclear factor kappa-light-chain-enhancer of activated B cells, PI3K: phosphatidylinositol 3-kinase, AKT: protein kinase B, NLRC4: NLR family CARD domain-containing protein 4, MyD88: myeloid differentiation primary response gene 88, AP-1: activator protein 1, TLR: toll-like receptor, IL: interleukin, MIP: macrophage inflammatory protein, FFAR1: free fatty acid receptor 1, MCP1: monocyte chemotactic protein 1, TGF*β*: transforming growth factor beta, and TNF*α*: tumor necrosis factor alpha.

The foremost target organ after oleic acid intravenous inoculation is the lungs, which retains about 85% of free fatty acids. The initial lesions occur as early as 5 min after administration [37] and last at least for 24 hours [38]. Oleic acid injected into the lung also induces neutrophil accumulation [36]. Increased TNF*α* and IL-8 levels after oleic acid injection [39], as well as IL-6, IL-1*β*, and the chemokine MIP-1*α* [36], were reported. Hence, oleic acid induces the synthesis of the main inflammatory mediators involved in clinical ARDS (Table 3 and Figures 1 and 2).

Lipid bodies numbers are increased in cells involved in inflammatory and immunologic processes [40]. Lipid bodies function as privileged sites is generating the lipid mediators LTB_4_ and PGE_2_ [41]. Oleic acid intratracheal instillation augmented lipid body numbers and LTB_4_ [42] and PGE_2_ levels [42, 43]. The intravenous injection of oleic acid increased lipid bodies formation and increased PGE_2_ levels in bronchoalveolar fluid lavage (BALF) [38] (Table 3 and Figure 1). Remarkably, the rise of LTB_4_ and PGE_2_ in human samples preceded ARDS in injured blunt-trauma patients [44], indicating similar features between experimental models using oleic acid and clinical events. Also, similar to oleic acid-induced lung injury, hemorrhage can arise in severe ARDS seen in patients with complicated leptospirosis [36, 45] (Figure 1).

Pulmonary edema formation can represent a life-threatening situation if it is not properly removed. Oleic acid is a NKA inhibitor [46] and also a Na^+^ channel inhibitor in the lung [47] resulting in a significantly increased endothelial permeability. We developed an assay that may allow researchers to study the importance of NKA activity using OA and ouabain (a classical NKA inhibitor) as a prove-of-concept control [48] (Figure 1). We showed that oleic acid inhibited NKA* in vivo* by measuring the uptake of rubidium by lung tissue and further that oleic acid inhibition was similar to ouabain. This animal model can be used to assay NKA inhibition not only in oleic acid-induced lung injury but also when using other molecules [48].

The leptospiral component glycolipoprotein fraction (GLP) has cytotoxic activity, and oleic acid is a major component of GLP [49]. Furthermore, we showed that the GLP lipid content handles NKA inhibition indicating that oleic acid has a crucial role in NKA inhibition either alone or as a part of a macromolecular complex. Recently we showed that GLP induces lung injury similar to ouabain and oleic acid [50]. Thus, oleic acid prevents edema clearance and can trigger protein-rich edema formation by intravenous or intratracheal routes [36, 38].

*Intracellular Pathways Activated in Oleic Acid-Induced Lung Injury and Inflammation.* Oleic acid may trigger diverse intracellular pathways altering cell functions. Here we discuss critical pathways induced by oleic acid impacting on lung damage.

The protein phosphatase and tensin homologue deleted on chromosome Ten (PTEN) is a major suppressor of phosphatidylinositol 3-kinase (PI3K)/protein kinase B (Akt) signaling, a vital survival pathway in lung cells (Figure 2). PTEN inhibition by bpV(phen) increased lung tissue levels of phospho-Akt and ERK and reduced the severity of oleic acid-induced ARDS in mice [51] (Table 3). ERK pathway participates in chemoattractant-induced neutrophil chemotaxis and respiratory burst as well as in LPS-induced ARDS [52]. In alveolar macrophages, the combined inhibition of p38 and ERK1/2 induced suppression of cytokine release [53]. ERK1/2 inhibition blocked neutrophil migration, edema, lipid body formation, and IL-6 production in a mice model of oleic acid-induced lung injury [36].

The serine/threonine kinase mammalian target of rapamycin (mTOR) is a key signaling kinase linked to several cellular functions including immunological and inflammatory responses. The mTOR inhibition reduced inflammatory cytokines in LPS/oleic acid-induced lung injury model [54].

Apelin is a group of small peptides derived from a common precursor, preproapelin. All apelin peptides exert their biologic effects by binding to a G-protein-coupled receptor, the APJ receptor, leading to biologic responses [55]. The apelin and APJ receptor are upregulated during tissue injury [56, 57]. A recent report showed that the inhibition of apelin-APJ alleviated lung inflammation and injury and improved oxygenation in oleic acid-induced lung injury [58].

Cell damage caused by the direct binding of oleic acid to biological membranes may be pivotal in oleic acid-mediated lung injury. Oleic acid triggers intracellular pathways ending up in lung cells death. It is directly toxic to endothelial cells in the lung [37], causing necrosis and inducing capillary congestion and interstitial/intra-alveolar edema [35] (Figure 1). Oleic acid induces mainly necrosis, but it also provokes apoptosis through a decrease in the antiapoptotic marker Bcl-2 and a marked increase of proapoptotic marker Bad [59]. Oleic acid also activates caspases 3 and 6 (Figure 2), enhancing the generation of reactive oxygen species and inducing a significant mitochondrial depolarization and apoptosis in leukocytes [60–62].

Oleic acid may work as a particular chaperon that modulates the interaction of cardiac glycosides with the NKA and hence alteration of oleic acid plasma levels would alter ouabain effects [63]. The NKA interacts with different signaling proteins forming a protein complex called signalosome [64]. The NKA signal transduction and ion pump functions work in independent fashion [65]. The oleic acid-induced lung inflammation may start with NKA activation [38]. Oleic acid triggers intracellular pathways in the lung and here we reinforce the critical role of NKA for inflammation in oleic acid-induced lung injury.

## 8. Animal Models of Oleic Acid-Induced Lung Injury

Animal models of lung injury were developed in the attempt to mimic the human ARDS. ARDS animal models may help us to understand the mechanism of ARDS. Unfortunately, no animal model mimics human ARDS exactly. Nonetheless, animal's studies can bring up crucial elements of lung injury in humans. In this regard, animal models can serve as a bridge between patients and lab research.

The oleic acid model was developed as an attempt to reproduce ARDS due to lipid embolism [66]. Variation in the outcome of this model is likely a consequence of variation in the preparation of oleic acid infusion that could be avoided by injection of oleic acid in a salt form [42], avoiding unwanted effects of ethanol, DMSO, or fatty embolism caused during blood emulsification.

The oleic acid induces early, fast, and reversible sparse inflammatory lung injury with permeability alterations and deficiency in gas exchange and lung mechanics. One advantage of this model is its reproducibility. The oleic acid inoculation provides a superb model to study ventilatory strategies, lung mechanics, and ventilation/perfusion ratio distribution during lung injury in large and small animals [35, 39, 67].

One drawback is the requirement of expertise in intravenous administration in small animals like mice. Another possible downside of the model is the relevance in human disease of oleic acid-induced ARDS. Here we strongly advocate in favor of a high relevance lipid metabolism alteration linked to ARDS: in particular, but not limited to, cases of sepsis, severe leptospirosis, preeclampsia, and pancreatitis [32, 68–71]. Further, we showed oleic acid-induced lung injury in mice via pulmonary and extrapulmonary routes that are similar to ARDS [36, 38]. Additionally, ARDS patients presented elevated oleic acid levels in the blood and lung [68], and this fact supports the idea that oleic acid has a critical role in ARDS pathogenesis (Figure 1).

Even though none of animal models fully mimics findings in the human disease, studies using animal ARDS models endure a vital biological tool to study the pathophysiology of and to test novel therapeutic interventions. It is easily reproducible and reliable and, therefore, a powerful model to study lung injury mechanisms and putative candidates in the ARDS treatment.

## 9. Conclusion

Oleic acid-induced lung injury is a relevant model to study ARDS because this fatty acid acts directly on the lung cells or lung endothelium and triggers activation of different innate immune receptors. It leads to cell activation, inflammatory mediator production, and cell death (Table 3), thus closely mimicking human ARDS. Further, NKA inhibition by oleic acid likely plays a critical role in lung injury during conditions of high oleic acid plasma levels, such as sepsis, leptospirosis, pancreatitis, and preeclampsia. Several key features of ARDS could be explored in the animal model of oleic acid-induced lung injury. Oleic acid concentration in plasma and/or BALF of patients is a critical predictor of ARDS development or outcome and, therefore, it could be used as a biological marker of disease severity. Therefore, the oleic acid model is likely suitable for studying the pathophysiology of ARDS.

## Figures and Tables

**Table 1:** Major agents that cause pulmonary injury.

Cell type	Agents
Alveolar types I and II cells	Pulmonary aspiration (HCl), trauma, lung infection (alive microbes or microbes secreted molecules), smoke inhalation (tobacco and other molecules), oleic acid, LPS, drug overdose, and inflammatory mediators

Endothelial cells	Systemic infection (sepsis, alive microbes or metabolic products), oleic acid, LPS, fatty embolism, large volume blood replacement, burn injury, inflammatory mediators, and autoantibodies

**Table 2:** Functions of the main lung cell types affected in ARDS-lung injury.

Cell type	Functions
Alveolar type I cell	Majority of the alveolar surface coverage, alveolar-capillary barrier formation, alveolar fluid clearance, and gas exchange

Alveolar type II cell	Surfactant secretion, epithelial cell regeneration after injuries, alveolar-capillary barrier formation, alveolar fluid clearance, gas exchange, and inflammatory mediators formation

Endothelial cell	Alveolar-capillary barrier formation, gas exchange, and inflammatory mediators production

Alveolar macrophages	Danger-associated molecular patterns recognition, immune response triggering, and chemotactic and inflammatory mediators secretion

**Table 3:** Key features of oleic acid-triggered lung injury and inflammation.

	Oleic acid
Direct and indirect lung injury induction	x
Cytokine induction	TNF*α*, IL-6, and IL-1*β*
Chemokine induction	IL-8, MIP-1*α*
Cell death induction	Apoptosis, necrosis
Sodium potassium ATPase inhibition	x
Immune innate response receptor activation	GPRC, NKA signalosome
Hyaline membrane formation	x
Lung hemorrhage induction	x
Lung cell infiltration/accumulation	Neutrophil, mononuclear cells
Lung function impairment	x
Protein-rich edema formation	x
Time line, course of lung injury	5 min up to 24 h
Lipid body formation	x
Lipid mediator induction	PGE_2_, LTB_4_
Intracellular pathway activation	MAPK ERK1/2, PI3K/Akt, sPLA(2), caspases 3 and 6, apelin-13, and mTOR
